# Early BCG vaccine to low-birth-weight infants and the effects on growth in the first year of life: a randomised controlled trial

**DOI:** 10.1186/s12887-015-0452-2

**Published:** 2015-09-28

**Authors:** Sofie Biering-Sørensen, Andreas Andersen, Henrik Ravn, Ivan Monterio, Peter Aaby, Christine Stabell Benn

**Affiliations:** Research Center for Vitamins & Vaccines (CVIVA), Bandim Health Project, Statens Serum Institut, DK-2300 Copenhagen S, Denmark; Projécto de Saúde Bandim, INDEPTH Network, Codex 1004 Bissau, Guinea-Bissau; Odense Patient data Explorative Network, Institute of Clinical Research, University of Southern Denmark/Odense University Hospital, DK-5000 Odense C, Denmark

**Keywords:** Neonates, BCG, Vitamin A supplementation, Non-specific effects of vaccines, Low-birth-weight, Infant growth

## Abstract

**Background:**

Randomised trials have shown that early Bacille Calmette-Guérin (BCG) vaccine reduces overall neonatal and infant mortality. However, no study has examined how BCG affects growth. We investigated the effect on infant growth of early BCG vaccine given to low-birth-weight (LBW) infants.

**Methods:**

Two-thousand three hundred forty-three LBW infants were randomly allocated 1:1 to “early BCG” (intervention group) or “late BCG” (current practice). Furthermore, a subgroup (*N* = 1717) were included in a two-by-two randomised trial in which they were additionally randomised 1:1 to vitamin A supplementation (VAS) or placebo. Anthropometric measurements were obtained 2, 6, and 12 months after enrolment.

**Results:**

Overall there was no effect of early BCG on growth in the first year of life. The effect of early BCG on weight and mid-upper-arm circumference at 2 months tended to be beneficial among girls but not among boys (interaction between “early BCG” and sex: weight *p* = 0.03 and MUAC *p* = 0.04). This beneficial effect among girls was particularly seen among the largest infants weighing 2.0 kg or more at inclusion.

**Conclusion:**

Though BCG vaccination is not recommended to be given to LBW infants at birth in Guinea-Bissau, early BCG had no negative effect on infant growth and may have had a beneficial effect for girls.

**Trial registration number:**

ClinicalTrials.gov (NCT00146302).

**Electronic supplementary material:**

The online version of this article (doi:10.1186/s12887-015-0452-2) contains supplementary material, which is available to authorized users.

## Background

Childhood vaccines may have non-specific effects on overall mortality [1–10], i.e., effects that cannot be ascribed to protection against the targeted diseases. The Bacille Calmette-Guérin (BCG) vaccine has been shown to have beneficial effects on overall mortality not explained by protection against tuberculosis, as suggested by historical data from England when BCG was introduced [8], observational studies from West Africa [3–7] and most recently demonstrated in randomised trials [9, 10].

In Guinea-Bissau, normal-birth-weight infants receive BCG at birth. However, according to local policy in Guinea-Bissau and other Sub-Saharan countries low-birth-weight (LBW) infants (<2500 gr) only receive BCG when they have gained weight, typically when they come for their first diphtheria-tetanus-pertussis (DTP) vaccination recommended at 6 weeks of age. This has made it possible to test the effect of early versus late BCG on infant mortality in two randomised trials conducted from 2002 to 2008. In these trials, LBW infants were randomised to receive BCG at discharge from the maternity ward or, if delivered at home, at the first contact with a health centre after birth (“early BCG”, intervention group) versus the usual delayed BCG (“late BCG”, control group) [9, 10]. A combined analysis of these trials showed that early BCG was associated with a borderline significant reduction in infant mortality of 21 % (95 % CI:-2 %; 39 %), and a 48 % (95 % CI: 18 %; 67 %) reduction in neonatal mortality, before most children in the control group received BCG [10]. Most of the reduction in neonatal mortality was caused by a prevention of deaths from sepsis and respiratory infections [9].

If early vaccination with BCG reduces the risk of contracting infectious diseases or reduces the severity of the infectious diseases, it could promote childhood growth. On the other hand, if more frail children survive in the “early BCG” group, this could create a false positive association between early BCG and poor nutritional status. No study has investigated the effect of BCG on growth. We therefore used data from the larger of the two previous randomised trials to test the effect of providing early BCG to LBW infants on growth in the first year of life. A subgroup of children from this trial was enrolled in a two-by-two factorial trial where they were additionally randomised to neonatal vitamin A supplementation (VAS) or placebo [11]. The effect of neonatal VAS on growth within this subgroup has previously been analysed; we found no strong effect [12].

## Methods

### Study design and randomisation

The Bandim Health Project maintains a health and demographic surveillance system (HDSS) in Bissau, the capital of Guinea-Bissau. The present growth study was conducted within a randomised trial which had the primary objective to investigate the effect of early BCG on infant mortality. The trial has been described in detail elsewhere [9]. In brief, from November 2004 to January 2008 children born at the national hospital in Bissau city who were ready to be discharged and children born at home who came for their first vaccination at three local health centres were invited to participate provided they weighed less than 2500 g at the time point of contact. Mothers/guardians of eligible children were informed of the study in the local language, Creole, and received a written explanation of the study in the official language, Portuguese. Consent of the mother/guardian was given by signature or fingerprint. Provided consent, the mother/guardian drew a lot from a bag that ensured the child was randomly allocated to “early BCG” versus “late BCG”. Twins were assigned to the same treatment to prevent potential confusion in case one twin died. Children allocated to “early BCG” were vaccinated intradermally with 0.05 ml BCG vaccine (Statens Serum Institut, Copenhagen, Denmark). The children who were allocated to “late BCG” were treated according to local practice and hence not vaccinated. These children would be vaccinated at a local health centre when they had obtained a normal birth weight or when they came for their first DTP vaccination at 6 weeks of age. We obtained information about date of BCG vaccination in the control group from the health card which has no information about the strain of BCG used. The subgroup of the children (*N* = 1717) enrolled at the national hospital from May 2005 to January 2008 were furthermore randomised to neonatal vitamin A supplementation (VAS) or “placebo” at discharge from the hospital. VAS was 0.5 ml vegetable oil with 25,000 IU vitamin A and 10 IU vitamin E. The placebo was 0.5 ml of the same oil with 10 IU vitamin E (Skanderborg Apotek, Denmark).

The children and their mothers were driven home from the hospitals by the field team. The field team drew a map of the house, recorded GPS coordinates, and took a photograph of the house and the mother to ensure that the team would be able to localise the child at subsequent visits.

### Anthropometrics

Weight, length, head circumference and mid-upper-arm-circumference (MUAC) were measured by trained field assistants at enrolment and at the home visits scheduled 2, 6, and 12 months after enrolment. The weight of the undressed child was measured using an electronic scale (SECA Model 835) to the nearest 10 g. Length was measured with a measuring mat (SECA Model 210) while the child was lying down. Head circumference on the widest possible circumference was measured using non-stretchable measuring tape (SECA Model 212). MUAC was measured on the left mid-upper arm using a non-stretch insertion tape (TALC, St. Albans, UK).

If children were absent at the time of the home visit, an attempt was made to revisit them shortly afterwards. Children who were travelling were only visited at the following scheduled visit. When a child moved within the city of Bissau, a relative or a neighbour usually showed the field assistants to the new house to minimize the loss to follow-up. Children who moved outside the city of Bissau were considered lost to follow up.

### Statistical analysis

Measurements for weight, length and head circumference were converted to z-scores using the 2006 WHO reference standards [13]. The original scale in cm for MUAC was used since no reference standards exists for MUAC below 3 months of age. Furthermore, we have found that MUAC on the original scale is as good a predictor of mortality as MUAC z-score [14]. The effect of early BCG on growth was analysed at 2, 6, and, 12 months after enrolment. Chi-square test, *t*-test and Kruskal-Wallis test was used to compare the baseline characteristics of the intervention groups. We calculated curves made from nonparametric, locally weighted regression (lowess curves) to illustrate the patterns of growth for children in the study.

We used general/multivariate normal linear models estimated by maximum likelihood to examine the associations between early BCG and the anthropometric measurements across time taking into account correlation of measurements within children [15]. The model includes a time variable (time) and an interaction between early BCG and time (early BCG × time) allowing separate effects of early BCG to be estimated at 2, 6 and 12 months. The model adjusted for the baseline measurement at inclusion by including an interaction between baseline and time (baseline × time). Unstructured covariance matrices were used to keep variances and correlations unconstrained. Robust standard errors were used to calculate confidence intervals. The model is often called Multivariate- or Mixed Model Repeated Measures denoted MMRM. If Y_it_ denotes the follow-up measurement, Y_i0_ the baseline measurement, and G the randomisation group; α_t_, β_t_, γ_t_ represents the time, baseline, and group coefficients and ε_it_ the residuals, the model can be written as:

$$ {\mathrm{Y}}_{\mathrm{i}\mathrm{t}} = {\alpha}_{\mathrm{t}} + {\beta}_{\mathrm{t}}{\mathrm{Y}}_{\mathrm{i}0} + {\gamma}_{\mathrm{t}}\mathrm{G} + {\varepsilon}_{\mathrm{i}\mathrm{t}},\ \left({\varepsilon}_{\mathrm{i}1},{\varepsilon}_{\mathrm{i}2},{\varepsilon}_{\mathrm{i}3}\right) \sim \mathrm{N}\left(0,\varSigma \right),\ \varSigma = \left({\sigma_1}^2{\sigma}_{21}{\sigma}_{31}\right)/\left({\sigma}_{21}{\sigma_2}^2{\sigma}_{32}\right)/\left({\sigma}_{31}{\sigma}_{32}{\sigma_3}^2\right)\in \mathrm{M}\left(3,3\right) $$

In effect, this saturated simultaneous model of the measurements across time corresponds to three separate linear regression models with the 2, 6, or 12 months measurement as outcome and the baseline measurement as covariate. The main difference is that the correlation between the measurements at 2, 6, and 12 months is taken into account and all observed information is used.

There was a beneficial effect of early BCG on neonatal mortality as well as a reduction in infant mortality. The effect on infant mortality was strongest among the children with a weight below 1.50 kg at inclusion [9]. We suspected that this could influence the analysis of growth since more small and frail children might have survived in the early BCG group. This bias would therefore have the strongest effect for children with the lowest weight. We consequently conducted the analyses by weight at inclusion: <1.50 kg (low weight), 1.50–1.99 kg (medium weight), and 2.00–2.49 kg (higher weight).

Since the trial was partly a two-by-two factorial trial, we controlled for “VAS”, “Placebo”, or “Not randomised to VAS/Placebo” at birth, but this did not change the estimates and the variable was therefore not included in the final model. In our previous analysis of the neonatal VAS effects [12], we had tested for interactions between early BCG and VAS within the subgroup participating in the two-by-two factorial trial. For weight and head circumference BCG tended to be beneficial when given with VAS but not when given without VAS (interaction between “early BCG” and VAS: weight *p* = 0.06; head circumference *p* = 0.06). However, since the interactions were insignificant we present the results for the combined groups.

All analyses were stratified by sex because BCG might have sex-differential effects [2, 4, 16, 17].

Significance levels were 5 % and all tests were two-sided. Estimates were presented with 95 % confidence intervals. The statistical analyses were conducted in STATA version 12 (Stata Corporation, College Station, TX, USA).

### Ethics

The protocol was approved by The Gambia/MRC Scientific and Ethics committees, and the Guinean Ministry of Health’s Research Coordination Committee. The Danish Central Ethical Committee gave its consultative approval. All children invited to participate in the study were offered free consultations and essential drugs.

## Results

**Fig. 1:**
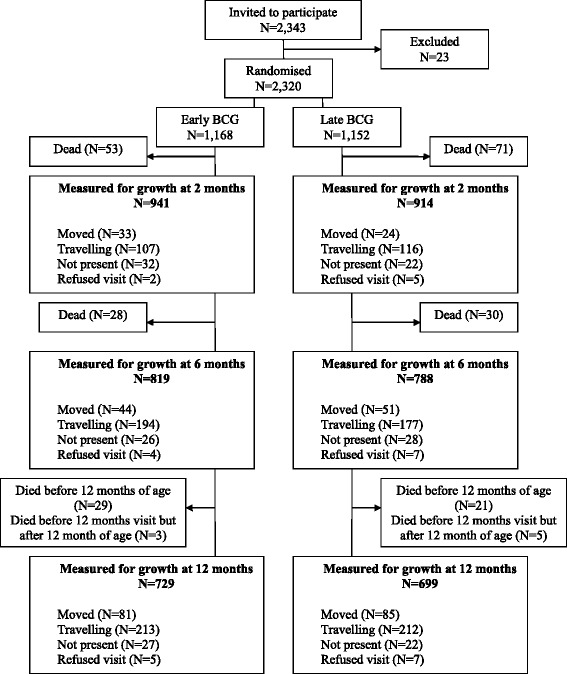
Flowchart

A total of 2343 children were invited to participate; of these 23 were excluded (Fig. 1). Hence 2320 children were randomised to early BCG or late BCG at inclusion. At baseline the early BCG and late BCG groups were comparable apart from the early BCG group having more twins/triplets and more mothers who were dead at enrolment [9]. The proportion of children that received OPV at birth were also comparable in the two randomisation groups. At 2 months, 465 children (total 20 %: early BCG:19 %/late BCG:21 %) were not examined anthropometrically, 713 children (total 31 %: early BCG:30 %/ late BCG:32 %) were missing at 6 months and 892 children (total 38 %: early BCG:38 %/late BCG:39 %) were missing at 12 months. The majority of children not examined were travelling or had died, with fewer deaths occurring in the early BCG group compared with the late BCG group (Fig. 1). Among the children seen at 2, 6 and 12 months of age, respectively, there were no baseline differences between the two randomisation groups (Additional file 1). Children never measured for growth at the follow-up visits had a lower weight and length, a smaller head circumference and MUAC as well as mothers with smaller MUAC at inclusion than children measured for growth (Additional file 2). In the intervention group, the median age of BCG vaccination was 2 days (10th–90th percentile: 1–10 days) (Additional file 1). In the control group 58 % had received a BCG vaccine at the 2 months visit [9] and the median age of vaccination was 47 days (20–57 days) (Additional file 1). At 12 months, 81 % of the children in the control group had received BCG [9] and the median age of vaccination was 49 days (22–99) (Additional file 1).

**Fig. 2:**
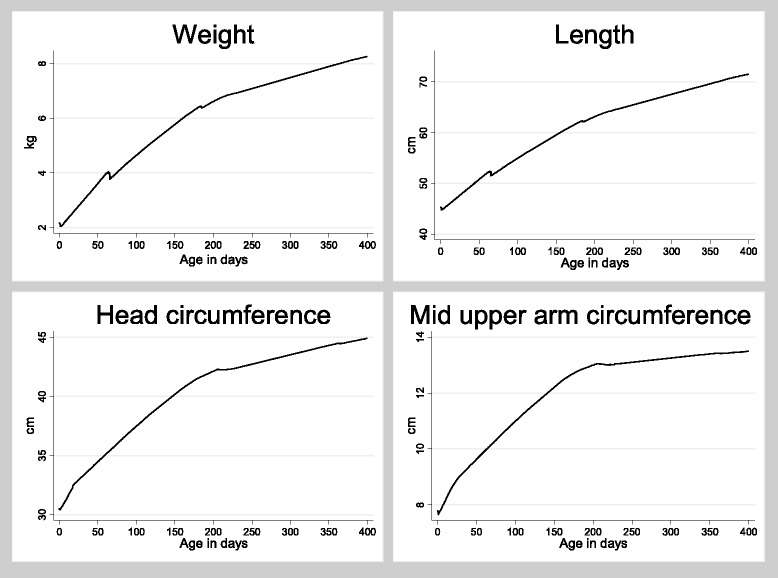
Lowess curves illustrating patters of growth for weight, length, head circumference and mid upper arm circumference (MUAC). The lowess curves are generated for both the early BCG group and the late BCG group together. There is no effect of BCG on the overall estimates why the lines for the two randomization groups could not be drawn separately

The patterns of growth for children in the study are presented in Fig. 2. The children showed the strongest increase in growth in the first 6 months of life. From 6 to 12 months the increase in growth slowed. This was most pronounced for MUAC.

### Overall effect of early BCG

**Fig. 3:**
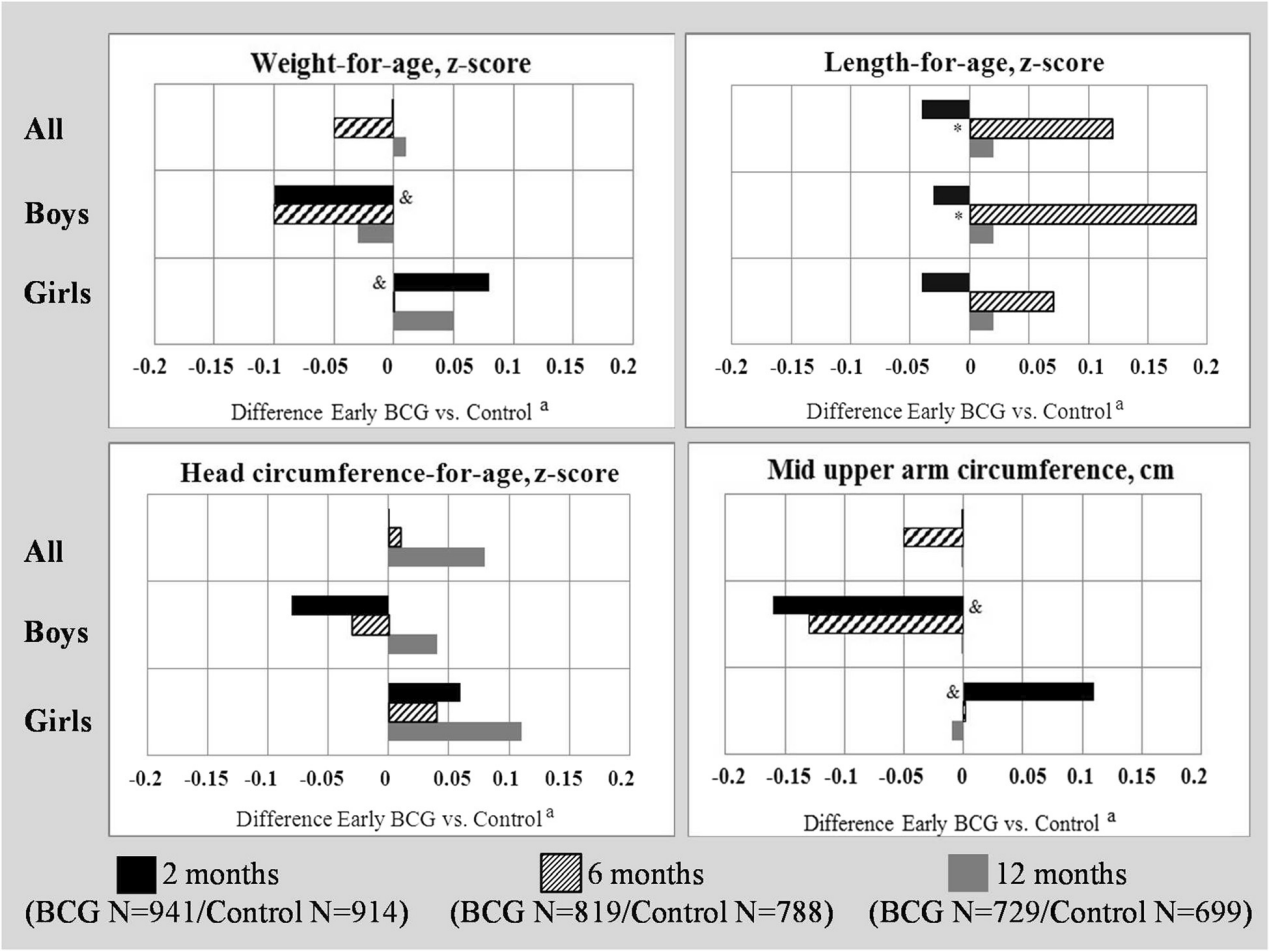
The effect of early BCG on anthropometric measurements at 2, 6 and 12 months. ^a^Analyses are conducted using longitudinal linear regression models containing information on the 2, 6 and 12 months measurements in one model. The analyses are furthermore adjusted for the corresponding measurement at enrolment. *Marks significant effect of early BCG (*p* < 0.05). & Marks significant interaction (*p* < 0.05) between sex and early BCG

At 2 months, there was no difference in weight, length, head circumference, and MUAC between the “early BCG” and “late BCG” groups (Fig. 3). At 6 months the children in the “early BCG” group had a higher length-for-age z-score (difference: 0.19 (CI95 %: 0.02; 0.37). No effects were seen for weight-for-age, head circumference-for-age, and MUAC. There were no differences between the “early BCG” and “late BCG” groups at 12 months for any of the anthropometric measurements.

Stratified by sex, there was a tendency towards a negative effect of early BCG for weight-for-age at 2 months among boys (−0.10 (−0.24; 0.04)) and a positive effect among girls (0.08 (−0.03; 0.20)) resulting in a significant interaction between early BCG and sex (interaction *p* = 0.04) (Fig. 3). A similar tendency was seen for MUAC at 2 months (boys: −0.16 (−0.34; 0.02) and girls: 0.11 (−0.03; 0.26)) likewise resulting in an interaction between early BCG and sex (interaction *p* = 0.04).

### Effect of BCG by weight at inclusion

Stratified by the three weight groups at inclusion, the effect of early BCG may have differed for weight-for-age and MUAC at 2 months (Table 1). The effect of early BCG on weight-for-age and MUAC tended to be beneficial in the highest weight group but the tendency was opposite in both the medium and the low weight group (high vs. medium/low: *p* = 0.04). When further stratifying by sex, the tendency towards a beneficial effect of early BCG in the highest weight group was only seen among girls for whom there was a significant beneficial effect of early BCG (weight-for-age: 0.15 (0.02; 0.28) and MUAC: 0.18 (0.02; 0.33)). Among boys there was a significant negative effect of early BCG on MUAC in the medium weight group (−0.39 (−0.77; −0.01)). There was a beneficial effect of early BCG on length-for-age at 6 months in the highest weight group (0.17 (0.05; 0.30)) and among girls in the highest weight group length was significant in its own right (0.20 (0.04; 0.37)) (Additional file 3).

**Table 1:** The effect of early BCG on anthropometric measurements at 2 months by weight at inclusion

	Weight at inclusion 2.00–2.49 kg			Weight at inclusion 1.50–1.99 kg			Weight at inclusion <1.50 kg		
	Mean^a^ early BCG	Mean^a^ late BCG	Difference^b^(CI: 95 %)	Mean^a^ early BCG	Mean^a^ late BCG	Difference^b^(CI: 95 %)	Mean^a^ early BCG	Mean^a^ late BCG	Difference^b^(CI: 95 %)
2 months									
All									
Number	643	646		242	216		56	52	
Weight-for-age, z-score	−1.58	−1.64	0.06 (−0.04; 0.16)	−2.97	−2.83	−0.14 (−0.34; 0.06)	−4.63	−4.53	−0.21 (−0.60; 0.18)
Length-for-age, z-score	−2.06	−2.03	−0.01 (−0.11; 0.09)	−3.45	−3.25	−0.16 (−0.37; 0.05)	−5.39	−5.37	0.02 (−0.42; 0.46)
Head circumference-for-age, z-score	−0.86	−0.90	0.02 (−0.09; 0.13)	−1.83	−1.92	0.06 (−0.14; 0.26)	−3.36	−3.49	−0.11 (−0.60; 0.38)
MUAC^c^, cm	11.6	11.5	0.07 (−0.06; 0.19)	10.5	10.6	−0.11 (−0.36; 0.13)	9.2	9.3	−0.20 (−0.71; 0.32)
Boys									
Number	284	278		106	100		21	21	
Weight-for-age, z-score	−1.80	−1.73	−0.05 (−0.22; 0.11)	−3.30	−3.03	−0.28 (−0.59; 0.03)	−4.97	−4.86	0.06 (−0.56; 0.68)
Length-for-age, z-score	−2.37	−2.24	−0.05 (−0.21; 0.11)	−3.81	−3.57	−0.17 (−0.46; 0.11)	−5.89	−5.87	0.51 (−0.12; 1.15)
Head circumference-for-age, z-score	−2.37	−2.24	−0.09 (−0.27; 0.08)	−2.05	−2.02	−0.02 (−0.34; 0.29)	−3.70	−3.99	0.04 (−0.66; 0.74)
MUAC^c^, cm	11.7	11.8	−0.08 (−0.28; 0.12)	10.4	10.8	−0.39 (−0.77; −0.01)	9.3	9.3	0.09 (−0.79; 0.97)
Girls									
Number	359	368		136	116		35	31	
Weight-for-age, z-score	−1.42	−1.56	0.15 (0.02; 0.28)	−2.71	−2.69	−0.03 (−0.29; 0.23)	−4.42	−4.30	−0.34 (−0.83; 0.15)
Length-for-age, z-score	−1.81	−1.87	0.04 (−0.09; 0.16)	−3.16	−2.97	−0.16 (−0.45; 0.13)	−5.09	−5.02	−0.32 (−0.90; 0.25)
Head circumference-for-age, z-score	−0.73	−0.84	0.12 (−0.03; 0.26)	−1.66	−1.84	0.12 (−0.14; 0.38)	−3.16	−3.15	−0.17 (−0.76; 0.43)
MUAC^c^, cm	11.6	11.4	0.18 (0.02; 0.33)	10.6	10.5	0.10 (−0.21; 0.41)	9.1	9.4	−0.29 (−0.88; 0.30)

^a^Unadjusted means

^b^Analyses are conducted using longitudinal linear regression models containing information on the 2, 6 and 12 months measurements in one model. The analyses are furthermore adjusted for the corresponding measurement at enrolment

^c^MUAC (Mid upper arm circumference)

## Discussion

### Main observations

There was no overall effect of early BCG on growth in the first year of life among LBW infants. Early BCG tended to be beneficial at 2 months for girls, but not boys, with respect to weight and MUAC. This beneficial effect among girls was particularly seen among the biggest infants weighing 2.0 kg or more at inclusion.

### Consistency with previous findings

No prior studies have examined whether BCG has an effect on growth. The present study indicated a beneficial effect of early BCG on growth during the first months of life for girls but not for boys, in line with previous observational studies which have shown a more beneficial effect of BCG on mortality for girls [2, 4, 16, 17].

### Strengths and weaknesses

We used data from a randomised controlled trial where follow-up was based on home visits. Only 12 % of the children were never measured for growth, mainly because they had already died or moved. The children never measured for growth were smaller than children measured for growth, which could be caused by more small children dying in the first month of life. Furthermore, the beneficial effect of early BCG on survival was most pronounced among children with a very low weight at inclusion [9] which could have masked a potential beneficial effect of early BCG on growth. We did adjust for the anthropometric measurement at inclusion. Hereby, we removed the effect of any baseline differences in anthropometric measurements between the intervention groups among children measured at 2, 6 and 12 months caused by more children dying in the late BCG group, and we also conducted the analysis stratified by weight at inclusion. However, if children with low growth-potential survive in the early BCG group and not in the late BCG group, it could still create a bias that cannot be corrected by baseline adjustment. The data did support this possibility since the beneficial effect was mostly seen in the children who weighed most at inclusion.

The trial was not blinded for ethical reasons; if we had used placebo mothers of control children might have believed that their child had already received BCG and hence not sought the vaccination later. The baseline measures were obtained before randomisation. The field assistants responsible for follow-up were not present at the time of randomisation, and though they could have actively sought the information from the mother, there were two assistants involved in all measurements and we find it unlikely that they were manipulated.

The children in the intervention group received the Danish strain of BCG (SSI, Denmark). Systematic information on BCG strain was not available in the control group; however, observation from the health centres suggests that they are most likely to have received the Russian strain. Some immunological studies have suggested that the Danish BCG strain may produce stronger beneficial non-specific effects compared to other strains of BCG [18]. Hence, the comparison of growth between early BCG and control groups may therefore have been biased by the intervention group receiving a BCG strain with stronger non-specific effects.

Most control children (58 %) had received BCG when they were measured at 2 months of age. Hence, the study did not assess the biological effect of BCG versus no BCG, but rather the effect of giving BCG early to all LBW children.

When stratifying the data on weight and sex, we perform a large number of subgroup analyses and hereby a large number of statistical tests. Due to the potential bias caused by the reduction in mortality from receiving an early BCG vaccine especially in the lowest weight group we believe it is necessary to conduct the weight-stratified analyses, though this obviously increases the risk of chance findings. In the weight-stratified analyses, however, the findings for weight-for-age, head circumference-for-age and MUAC all showed the same tendencies across weight groups making it unlikely to be chance findings.

### Interpretation

There were no strong effects on growth of receiving early BCG. One interpretation might be that early BCG does not affect growth. Alternatively, early BCG might have a beneficial effect on growth but due to the difference in mortality among the two intervention groups, this effect could be masked. The weight-stratified analysis lends some support to the latter interpretation. Since the relative difference in survival was less among children with a larger weight, growth among these children would more accurately show the effect of early BCG. In the highest weight group, early BCG had a beneficial effect; the effect on weight-for-age and MUAC being significant for girls. Hence, BCG might have beneficial effects on growth especially for girls.

## Conclusion

The present study found no overall effect on growth in the first year of life of providing early BCG to LBW infants. The study could not establish with certainty whether the results reflect lack of any effect of BCG on growth or it is due to the better survival of frail children in the early BCG group.

## Additional files

Additional file 1:
**Baseline differences among children measured for growth at 2, 6 and 12 months.** Description of the data: A table showing the baseline difference in the randomisation groups among the children measured for growth at 2, 6 and 12 months after inclusion. (XLSX 14 kb) Additional file 2:
**Baseline differences in children measured for growth and children never measured for growth.** Description of data: A table comparing baseline differences for children measured for growth and children never measured for growth. (XLSX 13 kb) Additional file 3:
**The effect of early BCG on anthropometric measurements at 6 and 12 months stratified by weight at inclusion, overall and by sex.** Description of the data: A table showing the effect of early BCG on anthropometric measurements at 6 and 12 months after inclusions stratified by weight at inclusion (2.00–2.49; 1.50–1.99; <1.50) and sex. (XLSX 12 kb)
